# Comparison of Sepsis-Induced Transcriptomic Changes in a Murine Model to Clinical Blood Samples Identifies Common Response Patterns

**DOI:** 10.3389/fmicb.2012.00284

**Published:** 2012-09-14

**Authors:** Sandro Lambeck, Martina Weber, Falk A. Gonnert, Ralf Mrowka, Michael Bauer

**Affiliations:** ^1^Integrated Research and Treatment Center – Center for Sepsis Control and Care, Jena University HospitalJena, Germany; ^2^Department of Anaesthesiology and Intensive Care Therapy, Jena University HospitalJena, Germany; ^3^Department of Internal Medicine III – Experimental Nephrology, Jena University HospitalJena, Germany

**Keywords:** innate and adaptive immune response, major histocompatibility complex class II, sepsis, systems biology, transcriptomics

## Abstract

Experimental models, mimicking physiology, and molecular dynamics of diseases in human, harbor the possibility to study the effect of interventions and transfer results from bench to bedside. Recent advances in high-throughput technologies, standardized protocols, and integration of knowledge from databases yielded rising consistency and usability of results for inter-species comparisons. Here, we explored similarities and dissimilarities in gene expression from blood samples of a murine sepsis model (peritoneal contamination and infection, PCI) and patients from the pediatric intensive care unit (PICU) measured by microarrays. Applying a consistent pre-processing and analysis workflow, differentially expressed genes (DEG) from PCI and PICU data significantly overlapped. A major fraction of DEG was commonly expressed and mapped to adaptive and innate immune response related pathways, whereas the minor fraction, including the chemokine (C–C motif) ligand 4, exhibited constant inter-species disparities. Reproducibility of transcriptomic observations was validated experimentally in PCI. These data underline, that inter-species comparison can obtain commonly expressed transcriptomic features despite missing homologs and different protocols. Our findings point toward a high suitability of an animal sepsis model and further experimental efforts in order to transfer results from animal experiments to the bedside.

## Introduction

Application of high-throughput technologies like microarrays extended the understanding of the complex molecular interactions in infectious disease processes (Jenner and Young, 2005; Polpitiya et al., 2009). Contrary to a single gene study, unbiased quantitative insights are collected by simultaneously monitored gene expression levels. These can be subject to data- and knowledge-driven analyses by bioinformatic and systems biology approaches in order to extract hypotheses about molecular interactions and predict interventional targets. Validation of these hypotheses may lead to translational approaches and ultimately optimize clinical practice (Cavaillon and Adib-Conquy, 2006). A major precondition for this comprises the inter-species comparison of molecular features in response to disease including their interactions as well as the underlying dynamics, which are covered by translational systems biology (Vodovotz et al., 2008). Experiments in animal models like *M. musculus* are often used to study human gene expression based on the assumption that the expression and function are similar for the majority of orthologous genes between both species (Lu et al., 2009). Although >12,000 orthologous gene pairs, i.e., homologous genes in the same syntenic location, were found in human and mouse, genomic differences with transcriptional and regulatory relevance exist (Mestas and Hughes, 2004). Comparison of expression differences in meta-studies, focusing on comparing lists of differentially expressed genes (DEG) derived from literature, e.g., studies about dietary restriction spanning six organisms for different species (Han and Hickey, 2005) or of sepsis markers in humans (Tang et al., 2010), yielded no agreement or only small intersections, respectively. This may be related to biological variability as well as different protocols for experimental set-up and data analysis. In contrast, promising transcriptomic comparisons from murine and human samples with respect to conserved pathways (Lim et al., 2010) as well as similar expression of homologs (Dowell, 2011) have been reported. Using a consistent workflow and filtering according to microarray quality control (MAQC, Shi et al., 2006) criteria, DEG with approximately 50% overlap in murine and human monocytes were obtained (Ingersoll et al., 2010), which is driving attempts to find conserved markers for diseases like sepsis across species.

Sepsis is a syndrome characterized by a systemic inflammatory response to infection (Bone et al., 1992) with high incidence in the intensive care unit (ICU) and especially critical for the Pediatric Intensive Care Unit (PICU) patients (Kovarik and Siegrist, 1998). The most severe form of sepsis, septic shock, is characterized by hypotension or hypoperfusion despite adequate fluid resuscitation. Therapies aimed at reducing the inflammatory burden of acute sepsis patients have been studied in clinical trials with little success, underlining the need to revise current approaches including the basic research in animals (Marshall, 2008). Experimental approaches in mimicking the sepsis syndrome include the use of peritonitis models like the polymicrobial challenges by peritoneal contamination and infection (PCI; Gonnert et al., 2011) and cecal ligation and puncture (CLP; Hubbard et al., 2005) or pneumonia models (Weber et al., 2012) aiming to closely reflect the pathophysiology observed in humans in the rodent models (Rittirsch et al., 2008).

In the present study, we investigated inter-species comparability based on gene expression patterns from experimental and clinical samples from septic blood. We compared time-resolved transcriptomic data obtained from (I) a murine polymicrobial sepsis model induced by PCI (Gonnert et al., 2011) and (II) sepsis and septic shock patients from the PICU and identified genes and pathways which could be translationally (across species) promising for diagnostics and interventions.

## Materials and Methods

### Data characteristics

Table 1 is summarizing the animal (I, PCI) and human (II, PICU) sepsis data used in this comparison further described in detail below.

(I) Animal experiments were carried out after approval of the local ethics committee. Using a recently published rodent sepsis model (Gonnert et al., 2011), 8-week-old female C57/BL6 mice were subject to the PCI challenge. Eight mice, equally divided into 6 and 24 h observational groups, received an intraperitoneal injection of 200 μl of a human feces suspension (0.21 × 10^6^ CFU/g, predicted to be lethal). Control mice (*n* = 4) were used for adjustments. Blood was drawn with a 20G Sterican needle (Braun, Melsungen, Germany) from the *Vena cava caudalis* in a syringe prepared with 200 μl PAXgene reagent (PreAnalytix^®^, Qiagen, Hilden, Germany). Clearing from globin RNA from whole blood samples was done by the *GLOBINclearTM Mouse/Rat Kit* by Ambion^®^ (Applied Biosystems, Darmstadt, Germany). RNA integrity and concentration analyses were performed with the Bioanalyzer 2100 (Agilent Technologies^®^, Palo Alto, CA, USA) according to the manufacturers specifications (*RNA 6000 Nano Assay*) using *RNA 6000 Nano LabChip*^®^ *Kits* by Agilent Technologies^®^. Amplification and biotinylation of RNA was in agreement to the protocol of the *Illumina*^®^ *TotalPrep RNA Amplifikation Kit* from Ambion (Applied Biosystems, Darmstadt, Germany). Hybridization was in accordance to the *Whole-Genome Gene Expression with IntelliHybTM Seal* protocol (Revision B) from Illumina^®^ (San Diego, CA, USA). Scanning of arrays was done with the BeadArray Reader 500 X for Illumina MouseWG-6 v2.0 expression beadchips. Validation of expression changes for candidate features PCI (6 h) was performed using a lower dose (0.14 × 10^6^ CFU/g, predicted to be lethal) for *n* = 2 gender- and age-matched mice monitored on Illumina MouseRef-8 V2 chips and compared to a control in a follow-up run.
(II) A publically available data set containing gene expression values from blood samples of PICU sepsis and septic shock patients and controls, along protocols as described elsewhere (Wong et al., 2009). Briefly, pediatric patients with septic shock were characterized by a significantly higher illness severity, higher mortality (*n* = 27 died within 28 days), and an increased degree of organ failure, compared to sepsis patients. Total RNA was isolated from whole blood samples using the PaxGene Blood RNA System according to the manufacturer’s specifications. Raw data for gene expression profiling was obtained from *Gene Expression Omnibus* (accession ID GSE13904).

**Table 1:** **Characteristics of transcriptomic data used in this analysis**.

Organisms (age)	PCI (experimental sepsis)	PICU (clinical sepsis and septic shock)	
	Female C57BL/6 mice (8-week old)	Pediatric intensive care unit (PICU) patients (2–5-year old)	
Syndrome	Sepsis	Sepsis	Septic shock
Case number [time (h)]	4, 4 (6, 24)	32, 20 (24, 72)	67, 39 (24, 72)
Controls	4	18	
Chip	Illumina Mouse WG 6 v2	Affymetrix HGU 133plus2	

### Data analysis

Computations were performed using R software (http://www.r-project.org/) version 2.15 and Bioconductor (Gentleman et al., 2004) packages. To assure data comparability (Ramasamy et al., 2008) a consistent workflow was applied.

(I) PCI data was subjected to quality control and the robust spline procedure from the R package *lumi* (Du et al., 2008), combining the features of quantile and loess normalization and log_2_ transformed signals were obtained. Bead types having detection values *p* < 0.01 in at least four of the RNA samples were taken. Subsequently, bead types were mapped to most recent annotation and gene-centered agglomeration by Entrez IDs was performed. Normalized detected bead types from PCI samples and controls are provided in Supplementary Tables 1 and 2, for the initial run and the validation data set, respectively.
(II) PICU data was pre-processed using chip definition file from *Brainarray* (v. 15.1, 2012) which aggregates probes into updated gene-centered probe set definitions mapped to Entrez IDs (Sandberg and Larsson, 2007). Further pre-processing was performed using quantile and loess normalization.

Entrez IDs were mapped using the Array Information Library Universal Navigator (AILUN)-service (Chen et al., 2007), facilitating cross-species and cross-platform comparisons by the use of homologous gene definitions. Merged data sets were re-normalized to avoid potential bias due to different log2 signal ranges. DEG were separately identified in PCI and PICU data according to micro array quality control (MAQC; Shi et al., 2006) – criteria and standard thresholds by: (1) average twofold difference for sample groups (opposed to controls) and (2) FDR (Benjamini and Hochberg, 1995)-adjusted *p*-values from moderated *t*-statistics <0.05 using *limma* (Smyth, 2004). Because different hybridization properties of the probes on species–specific arrays can profoundly bias cross-platform comparison of gene expression levels the use of relative expression changes is preferred to absolute expression signals (Liao and Zhang, 2006). Parametric tests (*t*-statistics) were applied to account for the small sample-sized PCI data. Pearson correlation (*r*) was used to assess similarity.

Hypergeometric tests were performed for the evaluation if intersections of separately identified DEG from PICU and PCI data could occur by chance (Fury et al., 2006). In addition, Monte–Carlo simulations (MCS, 4,000 iterations) were employed to non-parametrically assess the significance of intersections for two and more groups by re-sampling procedures (Ramasamy et al., 2008). One-sided *p*-values were defined by the fraction of counts derived from the empirical distributions of randomly expected intersections being larger than the number of observed intersections from the original gene lists.

To include potential differences in PICU (sepsis) and PICU (septic shock), commonly expressed DEG, i.e., found in human and murine data at any time point, were obtained by: DEGC = {DEG(PICU sepsis) ∪ DEG(PICU septic shock)} ∩ DEG(PCI). Differences in signs of expression changes were assessed by sign (DEGC(PICU)) ≠ sign (DEGC(PCI)). Hierarchical clustering (Eisen et al., 1998) of average log2 fold change data was applied for features drawn in a heatmap. Gene set enrichment analysis (GSEA) was performed using the *Database for Annotation, Visualization, and Integrated Discovery* (DAVID, v6.7; Huang et al., 2009) and its internal variant of Fisher’s exact test for enrichment within the pathways defined by the *Kyoto Encyclopedia of Genes and Genomes* (KEGG; Kanehisa et al., 2008) for murine and human data and backgrounds.

## Results

### Differentially expressed genes significantly overlap in both species

**Figure 1:**
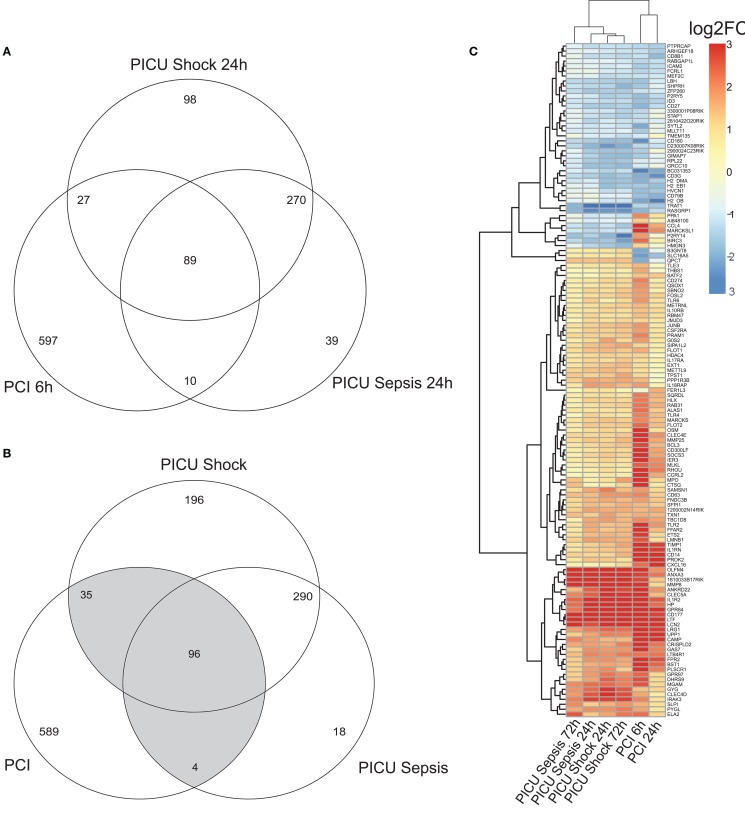
Venn diagram for **(A)** DEG for the early time point (with respect to controls) and **(B)** DEG for both time points for murine (PCI) and human (PICU) sepsis and septic shock data. **(C)** Heatmap for the 135 DEGC, i.e., genes, which are at least two-times differentially expressed (once in PCI and once in PICU). Features in the heatmap are annotated with gene symbols from *M. musculus* and average log2FC values (all adjusted to their respective controls) were pruned to be in [−3, 3].

Pre-processing yielded *k* = 18,988 and *l* = 9,325 gene-centered features from human and murine samples, respectively. Merging by Entrez IDs narrowed down the lists to *m* = 7,461 homologous features. Converted log2FC values (opposed to controls) of common detected features (*m*) indicated overall positive and significant correlations (*p* < 0.05) for PCI and PICU, but higher correlation within PICU data (*r* > 0.85). Combining DEG from PCI and PICU data as shown in the Venn diagrams for the early time point (Figure 1A) and both time points (Figure 1B) indicated time-persistent expression changes. Observed intersections of DEG from PCI and PICU were significantly higher than would be expected by chance (Figure 1A; e.g., *n*_1_ = 89 common DEG in PCI and PICU, *p* < 0.05 hypergeometric test; MCS sampling out of *m* features). Notably, septic shock induced more DEG than sepsis in PICU data (196 DEG were exclusively assigned to septic shock for both time points). DEGC, i.e., DEG found in PCI and PICU at any time point (Figure 1B highlighted in gray, *n*_12_ = 135, *p* < 0.05), as depicted in the heatmap (Figure 1C), indicated hierarchically grouped clusters for down- and upregulated genes. A small subset of DEGC (10 out of 135) was expressed with different signs, comprising, e.g., the chemokine (C-X-C motif) ligand 4 (*CCL4*) and *MARCKSL1* genes with high average disparity.

Assessing the dynamic changes for each group by the MAQC-criteria, e.g., PICU sepsis 72 h (late) *versus* PICU sepsis 24 h (early), yielded no qualitative changes in PICU sepsis data and only one gene in PICU septic shock data, but 141 features for PCI data (PCI 24 h *versus* PCI 6 h).

### Gene set enrichment analysis identifies pathways related to the innate and adaptive immune response

Next, we thought to determine the pathways exhibiting highest enrichment for the DEGC. Equally signed DEGC mapped to pathways from the immune response using DAVID knowledge base on KEGG (*M. musculus* definitions) as shown in Table 2.

**Table 2:** **Top five enriched KEGG pathways obtained from DAVID (*p*-values Fisher’s exact test *p* < 0.05) supported by FDR-adjusted *p*-values within the common down- and upregulated DEG from PCI and PICU**.

	Pathway	Hits	%	*p*-Value	FDR-adjusted *p*-value
Commonly downregulated genes (32 DAVID IDs)	Cell adhesion molecules (CAMs)	5	15.6	3.0E−04	7.7E−03
	Antigen processing and presentation	4	12.5	9.8E−04	1.3E−03
	Asthma	3	9.4	2.4E−03	2.1E−02
	Intestinal immune network for IgA production	3	9.4	6.3E−03	4.1E−02
	Allograft rejection	3	9.7	7.3E−03	3.7E−02
Commonly upregulated genes (87 DAVID IDs)	Cytokine–cytokine receptor interaction	7	8	2.0E−03	7.2E−02
	Insulin signaling pathway	5	5.7	6.8E−03	1.2E−01
	Toll-like receptor signaling pathway	4	4.6	1.7E−02	2.0E−01
	Jak-STAT signaling pathway	4	4.6	5.2E−02	4.0E−01
	Hematopoietic cell lineage	3	3.4	7.90E−02	4.7E−01

Top enrichment of commonly downregulated genes from the DEGC was found in the *CAMs*-category, containing *CD8B1*, *ICAM2*, and histocompatibility 2 class II locus genes (*H2-OB*, *H2-EB1*, and *H2-DMa*). Histocompatibility 2 class II locus genes were also included in the *Antigen processing and presentation* pathway and indicated an overlap for further categories, e.g., *Asthma*.

Top enriched category termed *Cytokine–Cytokine-Receptor Interaction* for commonly upregulated genes comprised chemokine (C-X-C motif) ligand 16, and related genes from interleukin receptors 1, 10, 17, and 18. Further enrichment was found for the *Insulin signaling pathway* (e.g., flotilin 1 and 2) and the Toll-like receptor (TLR) signaling pathway (*CD14*, *TLR2*, *TLR4*, and *TLR6*). Mapping of DEGC by human definitions/background to KEGG did not alter the ranking of enriched pathways.

### Validation of the transcriptomic response in murine sepsis

**Figure 2:**
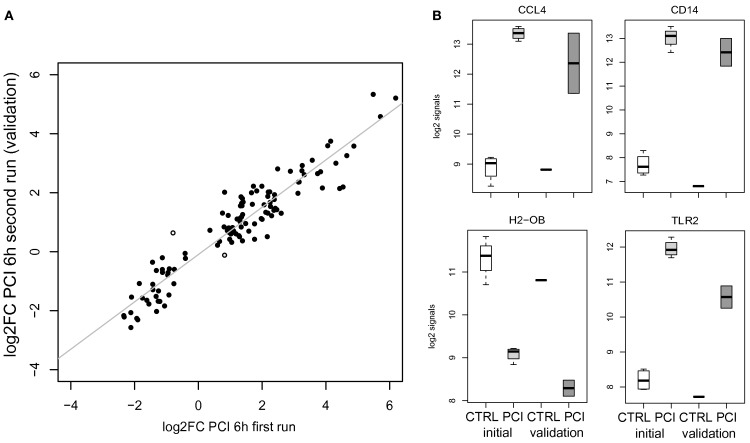
**(A)** Scatterplot opposing average log2FC values for DEGC (*n* = 115) in PCI for the 1st and 2nd run. Two spots with different signs for log2FC values are marked. **(B)** Gene-centered log2 signal values for controls (CTRL, *n* = 4), PCI 6 h (*n* = 4, light gray) of the initial run, and the validation run for control (CTRL, *n* = 1) and PCI 6 h (*n* = 2, gray).

Validation by a second run of wet lab experiments for the murine PCI challenge to a slightly lower dose yielded similar expression changes, i.e., significant positive correlation (*r* > 0.7, *p* < 0.05) by log2FC values of all detected genes on both Illumina platforms. Higher correlation (*r* > 0.9) was obtained for detectable DEGC (*n* = 115) in comparison of log2FC values of PCI data (first versus second run) as depicted in Figure 2A (including two highlighted outliers). Detailed log2 signals are exemplarily shown in Figure 2B for a selection of the aforementioned genes.

## Discussion

In the present work, we compared the transcriptomic response in blood samples from a murine sepsis model and pediatric septic patients. Batch-effects, due to different protocols, in this inter-species and cross-platform comparison, were approached by standard bioinformatic and microarray data analysis procedures including probe set re-annotation, group-wise control adjustments and using stringent MAQC-criteria for filtering of DEG. The analyzed spectrum of time-resolved gene expression data demonstrated positive correlations for PCI and PICU data with significantly overlapping DEG. Furthermore, commonly expressed genes were found throughout the observation period for both species, whereas the early time points (6 h PCI, 24 h PICU) already comprised the majority of identified DEG.

### Literature summary for genes and pathways related to the innate and adaptive immune response in sepsis

Although not statistically significant after multiple test correction commonly upregulated features mapped to *Cytokine–Cytokine-Receptor Interaction*, *Insulin signaling pathway*, and the *TLR signaling pathway*. The latter comprised *TLR2*, *TLR4*, and *TLR6* genes and can be grouped to the innate immune response (Tsujimoto et al., 2008). The differential regulation of these pathogen receptors is supported by a recent transcriptomic meta-study covering over 10 publications from human septic blood samples indicating a common activation of pathogen recognition receptors and corresponding signal transduction cascades (Tang et al., 2010).

Further features, which were identified commonly expressed in PCI and PICU, included the repressed MHC II genes, e.g., related to *Antigen processing and presentation*. Among many transcriptomic studies, MHC II related genes were found mostly stable downregulated and may characterize an immune paralysis (Prucha et al., 2004; Tang et al., 2009) related to reduced lymphocyte populations in sepsis due to apoptosis (Hotchkiss and Nicholson, 2006).

Furthermore a lot of unmapped features (not within KEGG pathway definitions for both species) were found and could be subject to enrichment tests in other knowledge bases. Comparison of DEGC to another published experimental sepsis study yielded, e.g., the significantly upregulated *LCN2* gene in the murine CLP model (Chung et al., 2006), whose protein mediating an innate immune response by sequestering iron (Flo et al., 2004).

Our results regarding the involved genes and pathways are supported by adult data from a recently published time-resolved transcriptomic study (Xiao et al., 2011), comparing a large cohort of severe trauma and burn injury patients to healthy subjects, where a sustained transcriptionally downregulated adaptive immune response (e.g., MHC II genes) as well as an upregulated innate immune response (including, e.g., *TLR*, *LTF*, *LCN2*, and *HP* transcripts) were found. As the degree of deregulation of these genes was able to discriminate between complicated and uncomplicated clinical courses, they might be of interest for novel diagnostic approaches in sepsis and other clinical entities.

### Limitations and perspectives

Limitations of our study include the differences in the wet lab protocols, because variation in the relative proportions of distinct cell types may contain valuable molecular information (Palmer et al., 2006). Furthermore, the number of homologous genes mapped by Entrez IDs across species may influence the results, as observed for the limiting PCI data set (*k*, *l*, *m*). Observed disparities in expression of the DEG, e.g., high average disparity in murine and pediatric sepsis samples for the *CCL4* and *MARCKSL1* genes, may reflect a bias within the mapping or the underlying genomic features. Because their expression can also be affected by missing homologs through complex interaction networks, experimental workarounds may include the use of transgenic or humanized mice (Shultz et al., 2007).

These limitations notwithstanding, we have done a first step, in comparing functional genomic-based experimental sepsis to pediatric patients data for potential modeling applications in translational systems biology. Future studies could also address the impact of different therapeutic approaches in sepsis across species, e.g., the response to antibiotics in combination with immunomodulators in PCI (Bauhofer et al., 2008) on the transcriptomic level.

## Conclusion

We found highly comparable gene expression patterns in blood samples from a murine sepsis model and pediatric septic patients characterized by commonly expressed genes from the adaptive and innate immune response. Findings point toward a high suitability of an animal sepsis model to study the complex molecular mechanisms and to establish diagnostic as well as treatment options for pediatric sepsis patients.

## Author Contributions

Sandro Lambeck performed the data analysis and provided access to computations to the reviewers, and drafted the manuscript. Martina Weber carried out the initial wet lab experiments. Martina Weber and Falk A. Gonnert participated in data interpretation. Ralf Mrowka and Michael Bauer supervised the work.

## Conflict of Interest Statement

The authors declare that the research was conducted in the absence of any commercial or financial relationships that could be construed as a potential conflict of interest.

## Supplementary Material

The Supplementary Material for this article can be found online at http://www.frontiersin.org/Microbial_Immunology/10.3389/fmicb.2012.00284/abstract
